# Stable ABTS Immobilized in the MIL-100(Fe) Metal-Organic Framework as an Efficient Mediator for Laccase-Catalyzed Decolorization

**DOI:** 10.3390/molecules22060920

**Published:** 2017-06-02

**Authors:** Youxun Liu, Yuanyuan Geng, Mingyang Yan, Juan Huang

**Affiliations:** 1 School of Basic Medical Sciences, Xinxiang Medical University, Jinsui Avenue 601, Xinxiang 453003, Henan, China; liuyouxun@126.com (Y.L.); 18790550067@139.com (Y.G.); mingyangyan@126.com (M.Y.); 2 Henan Collaborative Innovation Center of Molecular Diagnostics and Laboratory Medicine, Jinsui Avenue 601, Xinxiang 453003, Henan, China

**Keywords:** laccase, ABTS, metal-organic framework, mediator, decolorization

## Abstract

The successful encapsulation of 2,2′-azino-bis(3-ethylbenzthiazoline-6-sulfonic acid) (ABTS), a well-known laccase mediator, within a mesoporous metal-organic framework sample (i.e., MIL-100(Fe)) was achieved using a one-pot hydrothermal synthetic method. The as-prepared ABTS@MIL-100(Fe) was characterized by scanning electron microscopy (SEM), X-ray diffraction (XRD), Fourier transform infrared (FT-IR) spectroscopy, nitrogen sorption, and cyclic voltammetry (CV). Our ABTS@MIL-100(Fe)-based electrode exhibited an excellent electrochemical response, indicating that MIL-100(Fe) provides an appropriate microenvironment for the immobilization and electroactivity of ABTS molecules. ABTS@MIL-100(Fe) was then evaluated as an immobilized laccase mediator for dye removal using indigo carmine (IC) as a model dye. Through the application of laccase in combination with a free (ABTS) or immobilized (ABTS@MIL-100(Fe)) mediator, decolorization yields of 95% and 94%, respectively, were obtained for IC after 50 min. In addition, following seven reuse cycles of ABTS@MIL-100(Fe) for dye treatment, a decolorization yield of 74% was obtained. Dye decolorization occurred through the breakdown of the chromophoric group by the Laccase/ABTS@MIL-100(Fe) system, and a catalytic mechanism was proposed. We therefore expect that the stability, reusability, and validity of ABTS@MIL-100(Fe) as a laccase mediator potentially render it a promising tool for dye removal, in addition to reducing the high running costs and potential toxicity associated with synthetic mediators.

## 1. Introduction

Laccase (benzenediol oxygen oxidoreductase, EC 1.10.3.2) is an enzyme produced by various organisms, including fungi, bacteria, and plants [1]. It is an efficient and environmentally friendly catalyst for bioremediation, as it catalyzes the oxidation of phenolic compounds using molecular oxygen as an electron acceptor, producing only water as a byproduct [2]. As laccases have a broad range of substrates, their application in wood pulping, bioremediation, electrochemical analysis, and organic synthesis has received increasing attention in recent decades [2,3,4]. Although laccase cannot directly catalyze the oxidization of most nonphenolic substrates due to its relatively low redox potential, its substrate scope can be widened to nonphenolic compounds through combination with low molecular weight mediators, which simultaneously act as substrates for the enzyme [5,6]. For example, soluble redox mediators, such as diammonium 2,2′-azino-bis(3-ethylbenzothiazoline-6-sulfonate) (ABTS), 1-hydroxybenzotriazole (HBT), and TEMPO (2,2,6,6-tetramethyl-1-piperidinyloxy free radical) have been employed to enhance the oxidation capabilities of laccase towards nonphenolic compounds with high redox potentials [5,6]. In such cases, the mediator acts as an electron acceptor during the oxidation-reduction reaction, and participates in substrate oxidation. To interpret the mediator-substrate oxidation of laccase, a range of mechanisms have been proposed, including an ionic mechanism, two radical mechanisms, and electron transfer or hydrogen atom abstraction [7,8]. More specifically, mediators such as HBT and ABTS react via radical pathways, while other mediators including TEMPO and its analogues react through ionic pathways [7,8].

To promote the degradation of pollutants, the use of mediators is often recommended to improve the catalytic efficiency of laccase via the mediator-promoted enhancement of electron transfer [9]. Although both synthetic and natural mediators exist, the majority of natural mediators (e.g., syringaldehyde) have relatively low efficacies, and so the majority of currently available efficient mediators tend to be synthetic [3,5,9]. Although many studies have focused on reusability of enzymes in the laccase/mediator system, the recovery and reuse of such mediators has received little attention [5,9,10,11]. In addition, due to the high cost and potential toxicity of synthetic mediators for use in biocatalytic applications, their recovery and reuse is of particular importance. One previously reported technique to achieve this with enzymes is immobilization [12,13,14,15], which also has a number of economic and ecological merits. Indeed, the immobilization of various mediators using different methods has also been examined, where the coupling of TEMPO with polyethylene glycol allowed its reuse in azo dye decolorization by laccase [16]. In addition, a novel biocatalyst was prepared through the simultaneous immobilization of laccase and a mediator (i.e., acetylacetone) into a hydrogel via polymerization. This co-immobilized laccase/mediator system was them employed to enhance the biocatalytic transformation of malachite green [17]. Moreover, ABTS was cross-linked in a frozen aqueous poly (acrylate) mixture via electron irradiation, and the resulting immobilized redox mediators remained functional in accelerating the laccase-catalyzed degradation of BPA [18]. Furthermore, our group recently reported the use of ABTS-modified silica nanoparticles as mediators for a laccase-catalyzed dye decolorization [19]. However, the above mediator immobilization methods exhibit low immobilization efficiencies and require sophisticated techniques. As such, the development of a simple method to construct a support material for the immobilization of a mediator to facilitate its recovery and reuse remains a challenge.

In the last decade, the use of metal-organic frameworks (MOFs) in applications such as separation, catalysis, greenhouse gas storage, drug vectorization, and as contrast agents for magnetic resonance imaging, has received growing attention due to the unique features of these crystalline porous hybrid materials, including their high specific surface areas and tailorable pore dimensions [20,21,22,23]. Indeed, the tailorable pore sizes, compositions, and functionalizations of MOFs are of particular importance, as they allow the encapsulation of various kinds of molecules [22]. Furthermore, MOFs are also attractive host matrices for the encapsulation of catalysts to enable their facile recovery and reuse. For example, the encapsulation of phosphotungstic acid into MIL-101 by a one-pot synthetic method resulted in good catalytic performances in the dehydration of carbohydrates, in addition to facile catalyst recovery and recycling [24]. Alternatively, MIL-100(Fe) is an iron(III) polycarboxylate-based MOF, with a mesoporous three-dimensional cubic structure [25]. In terms of a potential mediator, ABTS is one of the most efficient redox mediators to act as an electron shuttle between the redox center of laccase and the substrate. In this context, it has been reported that the size of an ABTS molecule (i.e., ~6.4 × 6.4 × 17.4 Å) is highly compatible with the size of the hexagonal windows of MIL-100(Fe) (i.e., ~8.6 Å) [26], which could result in strong encapsulation of the mediator within the mesoporous cages of the MOF, thereby preventing rapid leaching. More importantly, MIL-100(Fe) exhibits long-term stability in water for up to two months [27], and so we expect that it will be an ideal matrix for the immobilization of ABTS.

**Figure 1:**
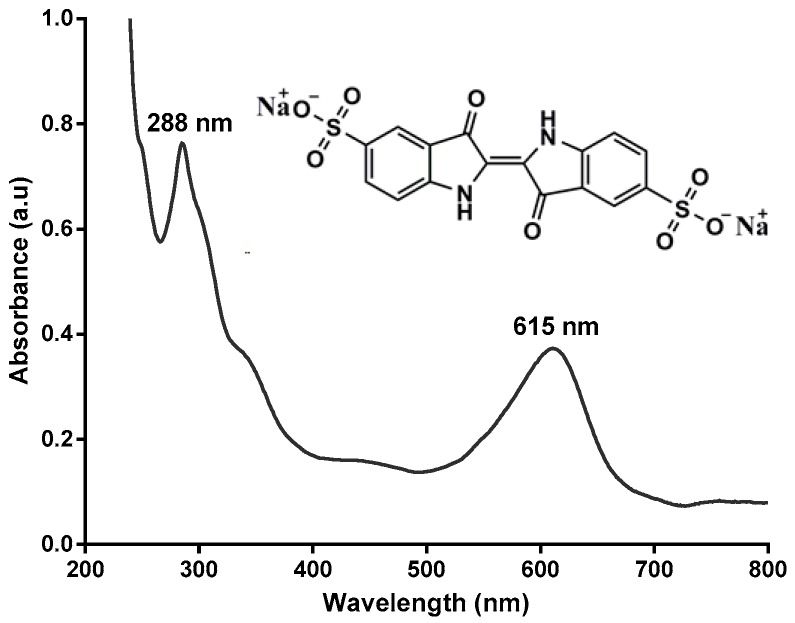
Molecular structure and UV-visible spectrum of indigo carmine.

As indigo carmine (IC) dye (Figure 1) is not a typical substrate of laccase, its complete decolorization by laccase requires the use of a mediator [19]. We therefore selected this dye as the model compound for the purpose of this study. Thus, we herein report the use of a one-pot synthetic method for the immobilization of ABTS in the pores of MIL-100(Fe). The resulting ABTS@MIL-100(Fe) hybrid materials will then be fully characterized by scanning electron microscopy (SEM), X-ray diffraction (XRD), Fourier transform infrared (FT-IR) spectroscopy, nitrogen sorption, and cyclic voltammetry (CV). Finally, we intend to use the MIL-100(Fe)-immobilized ABTS as a laccase mediator for dye decolorization and subsequent evaluation of its recovery and reuse.

## 2. Results

### 2.1. Synthesis and Characterization of ABTS @MIL-100(Fe)

**Figure 2:**
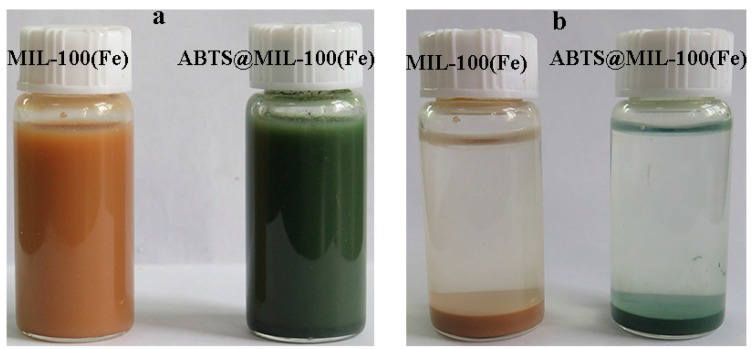
Photographic images of (**a**) suspensions and (**b**) precipitates of MIL-100(Fe) and ABTS@MIL-100(Fe).

Following synthesis of the desired nanoparticles, stable dispersions of MIL-100(Fe) and ABTS@MIL-100(Fe) were prepared in aqueous solution (Figure 2). As shown, the suspension of MIL-100(Fe) is orange, while that of the as-synthesized ABTS@MIL-100(Fe) is green, indicating that ABTS^+•^ radical cations are present in MIL-100(Fe). Furthermore, after resting overnight, a green precipitate was observed for the latter suspension, although the supernatant was colorless, thereby suggesting that the ABTS^+•^ radical cations were encapsulated in the MIL-100(Fe) matrix rather than being present in solution.

**Figure 3:**
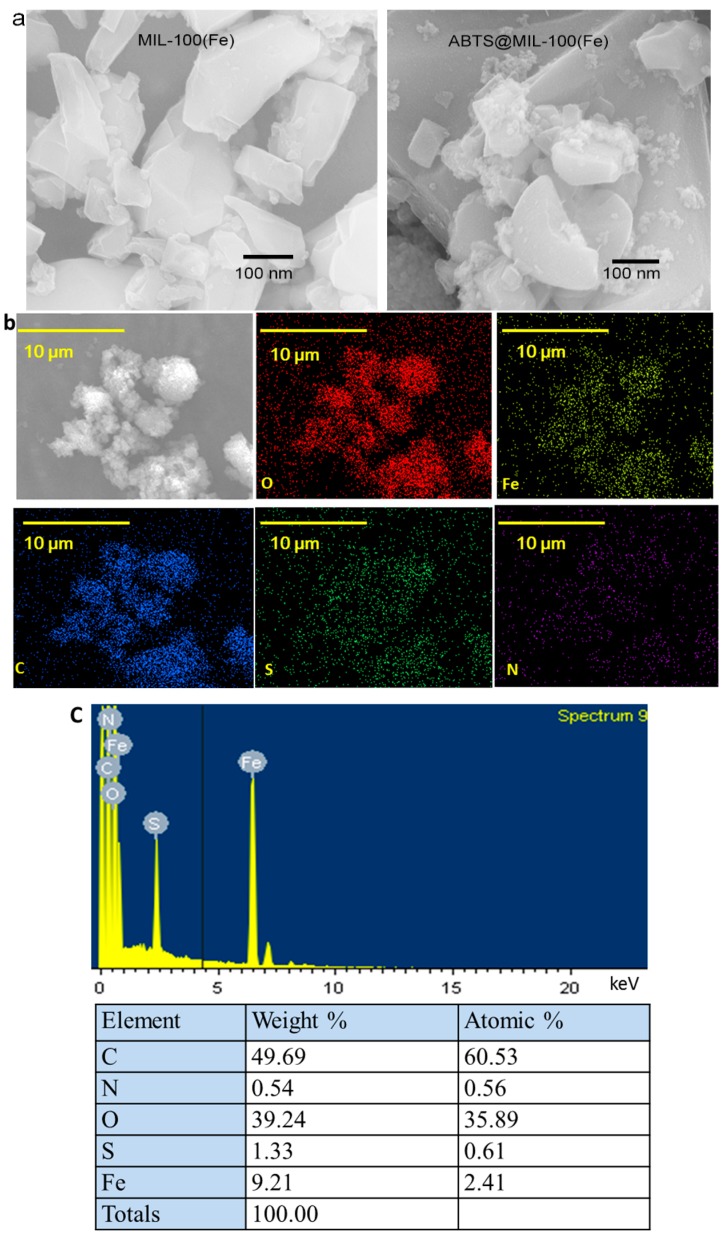
(**a**) SEM images of MIL-100(Fe) (left) and ABTS@MIL-100(Fe) (right); (**b**) SEM-mapping images for the distribution of different elements on the ABTS@MIL-100(Fe) surface; (**c**) EDS point spectrum and relative corresponding elemental contents.

The aggregations of MIL-101(Fe) and ABTS@MIL-100(Fe) were observed in the SEM images (see Figure 3a). When compared with the original MIL-100(Fe) particles, no major morphological changes were observed for the MOF following ABTS encapsulation. In addition, the SEM-mapping images shown in Figure 3b confirm the presence of O, Fe, and C in the MIL-100(Fe) framework in addition to the introduction of S and N during the ABTS encapsulation. Furthermore, the dispersion of S and N on the surfaces of the synthesized MOFs indicates that ABTS had been homogeneously loaded onto the pores of the MOF. Thus, based on the obtained energy spectra (Figure 3c), the chemical composition of the synthesized material was determined by energy dispersive spectroscopy (EDS), which confirmed that C, S, N, O and Fe were present in the ABTS@MIL-100(Fe) nanoparticles.

**Figure 4:**
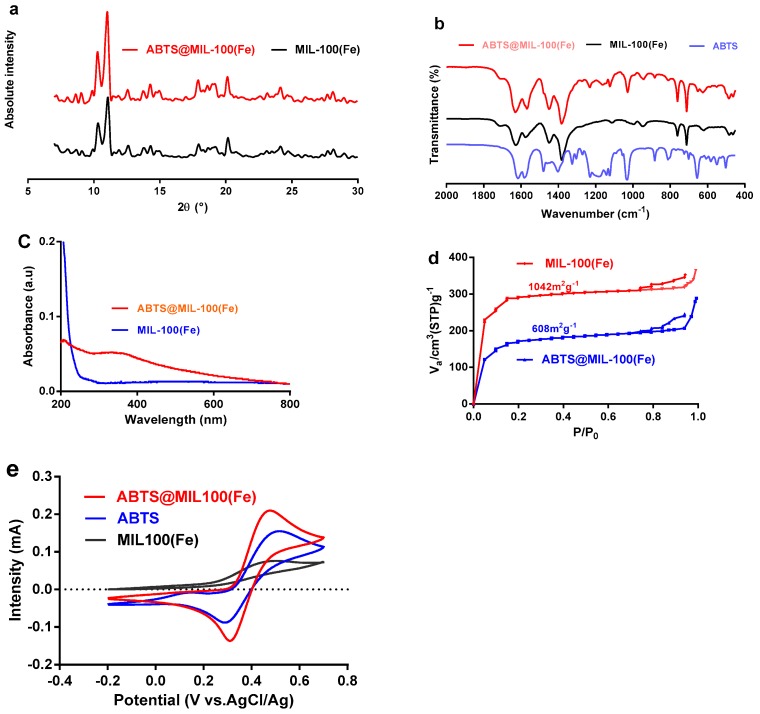
Characterization of the MIL-101(Fe) and ABTS@MIL-100(Fe) nanoparticles. (**a**) X-ray diffraction patterns; (**b**) Fourier transform infrared spectra; (**c**) UV-Vis absorption spectra; (**d**) N_2_-adsorption isotherms; (**e**) Cyclic voltammograms of the MIL-101(Fe), ABTS, and ABTS@MIL-100(Fe) modified Pt electrode at a sweep rate of 20 mV s^−1^ in 0.05 M acetate buffer at pH 5.5.

Subsequent analysis by XRD confirmed that the pattern obtained for the ABTS@MIL-100(Fe) composite is fully consistent with that of the parental MIL-100(Fe), indicating that the crystalline structure of MIL-100(Fe) was maintained following ABTS encapsulation (Figure 4a). The formation of ABTS@MIL-100(Fe) assemblies was further supported through comparison of the FT-IR spectrum of ABTS@MIL-100(Fe) with those of ABTS and MIL-100(Fe) (Figure 4b), as the spectra of ABTS@MIL-100(Fe) contained the characteristic bands of both ABTS and MIL-100(Fe). For example, the FT-IR spectrum of ABTS@MIL-100(Fe) displays signals at 1122, 1022, 872, and 655 cm^−1^ that correspond to the stretching and bending modes of the sulfonate groups on ABTS [28]. Furthermore, in the UV-visible (UV-Vis) absorption spectra of MIL-101(Fe) and ABTS@MIL-100(Fe) presented in Figure 4c, an absorption signal was observed at 340 nm for ABTS@MIL-100(Fe), which was attributed to the unsaturated double bonds of the ABTS molecule [29]. The permanent porosities of both MIL-101(Fe) and ABTS@MIL-100(Fe) were then verified by N_2_-adsorption isotherms (Figure 4d). Based on these results, the Brunauer-Emmett-Teller (BET) surface area of ABTS@MIL-100(Fe) (i.e., 608 m^2^ g^−1^) was found to be lower than that of MIL-101(Fe) (i.e., 1042 m^2^ g^−1^), which was attributed to the insertion of significant quantities of ABTS into the pores of MIL-100(Fe).

We then examined the electroactivity of the ABTS@MIL-100(Fe) nanoparticles by cyclic voltammetry using an ABTS@MIL-100(Fe)-modified Pt electrode at pH 5.5 in acetate buffer (Figure 4e). As indicated, the ABTS@MIL-100(Fe)-modified Pt electrode exhibited similar behavior with an ABTS-modified Pt electrode, while the MIL-100(Fe)/Pt electrode produced a very low redox peak. Indeed, the redox peaks observed between −0.20 and 0.60 V for the ABTS@MIL-100(Fe)/Pt electrode corresponded to the one-electron reversible redox process of ABTS [30,31].

### 2.2. Effects of pH on Dye Decolorization by Laccase with Either the Free or the Immobilized Mediator

**Figure 5:**
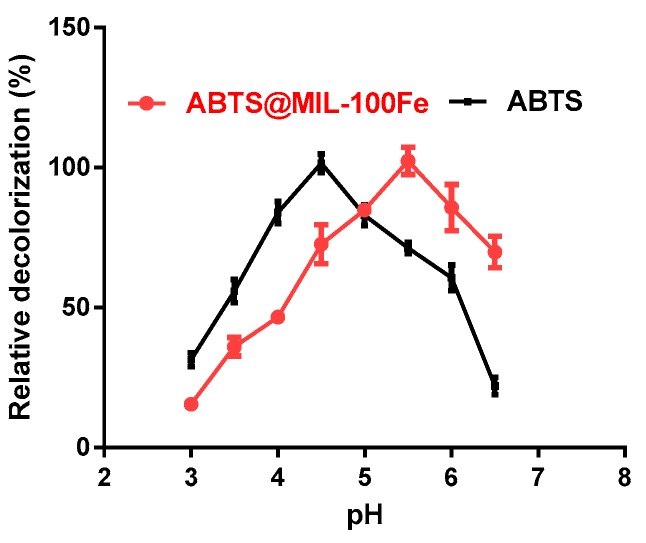
Effect of pH on dye decolorization by laccase using either ABTS or ABTS@MIL-100(Fe) in acetate buffer between pH 3.0 and 6.5.

The effect of pH on dye decolorization by laccase using either the free mediator (ABTS) or the immobilized mediator (ABTS@MIL-100(Fe)) was then determined in acetate buffer between pH 3.0 and 6.5 (Figure 5). As shown in the figure, the optimum pH for maximum decolorization by laccase in the presence of ABTS was pH 4.5, likely due to the laccase enzyme activity reaching a maximum when ABTS was used as the substrate [19]. However, the optimum pH for dye decolorization by ABTS@MIL-100(Fe) was pH 5.5.

### 2.3. Comparison of Dye Decolorization Efficiencies

**Figure 6:**
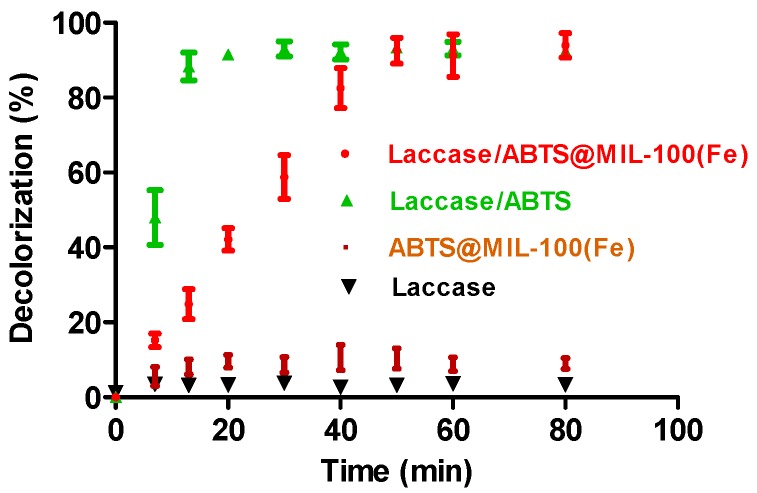
Dye decolorization by the laccase/mediator system, ABTS@MIL-100(Fe), and laccase alone as a function of time. The decolorization conditions were as follows: 50 mg/L dye, 2000 U/L laccase, an immobilized mediator (0.25 g/L for ABTS@MIL-100(Fe)) at pH 5.5 or a free mediator (25 µM for ABTS) at pH 4.5 (in 0.05 M acetate buffer).

The effect of the ABTS@MIL-100(Fe) on the decolorization efficiency of laccase was then examined through comparison of the decolorization efficiency of the laccase/ABTS@MIL-100(Fe) system with those of the laccase/ABTS system, ABTS@MIL-100(Fe), and laccase alone (Figure 6). For ABTS@MIL-100(Fe), only ~8% of dye decolorization was achieved after 15 min, and this value remained relatively constant with increasing reaction time. This decolorization was attributed to the adsorption of the dye molecules by ABTS@MIL-100(Fe). Although the ABTS@MIL-100(Fe) nanocomposite is a characteristic mesoporous solid with a high surface area (i.e., 608 m^2^ g^−1^), its adsorption capacity towards the dye is low, likely due to the majority of the pores in ABTS@MIL-100(Fe) being occupied by ABTS molecules. For laccase alone, a negligible decolorization (5%) was observed, as the indigo carmine dye employed herein is not a typical laccase substrate [16,32,33,34,35,36,37]. These results therefore indicate that decolorization of this dye by the MOFs and by laccase alone was negligible. In contrast, almost complete decolorization (95%) was achieved within 10 min by laccase when ABTS was employed as a mediator, and ~94% decolorization was achieved within 50 min using ABTS@MIL-100(Fe) as a mediator. Such high decolorization efficiencies were therefore attributed to the effect of the mediator rather than the MOFs or laccase alone.

### 2.4. Reusability of ABTS@MIL-100(Fe)

**Figure 7:**
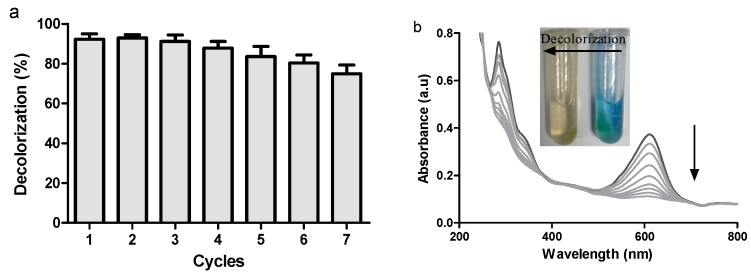
(**a**) Reusability of ABTS@MIL-100(Fe) (10 g/L) over 7 successive cycles using 2000 U/L laccase, and 50 mg/L indigo carmine in acetate buffer at pH 5.5, with shaking at 150 rpm for 50 min at 25 °C (**b**) UV-Vis spectra of the dye decolorization process using the laccase/ABTS@MIL-100(Fe) system at different time intervals. Also shown are photographic images of the samples both before and after decolorization.

We then examined the reuse and recycling of ABTS@MIL-100(Fe) as a mediator for dye decolorization over 7 cycles, as outlined in Figure 7a. With a reaction time of 50 min, comparably high decolorization yields were obtained over the seven runs using the recovered ABTS@MIL-100(Fe); however, the decolorization efficiency decreased gradually upon increasing the cycle number, with ~74% decolorization being obtained after the seventh cycle. Indeed, similar observations have also been reported by our group for the ABTS-SNP-catalyzed dye decolorization reaction [19]. In addition, the UV-Vis absorption spectra obtained at different time intervals during dye decolorization are shown in Figure 7b, where the absorption bands at 615 and 288 nm correspond to the blue color and unsaturated double bonds, respectively, in IC. As indicated, following treatment with the laccase/ABTS@MIL-100(Fe) system, this blue color decreased in intensity, and the solution eventually turned yellow/brown in color. As such, the disappearance of the absorbance peaks at 288 and 615 nm indicated that IC was successfully degraded by laccase in the presence of ABTS@MIL-100(Fe).

### 2.5. Stability Analysis

**Figure 8:**
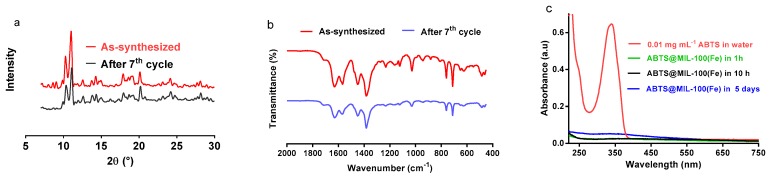
(**a**) X-ray diffraction patterns of ABTS@MIL-100(Fe) following preparation and after seven reuse cycles; (**b**) FT-IR spectra of the as-synthesized and recycled ABTS@MIL-100(Fe) samples; (**c**) UV-Vis spectra of ABTS@MIL-100(Fe) dispersed in water for 1 h, 10 h and 5 days. The spectrum of ABTS is also shown for comparison.

The stability and possible leaching of ABTS from MIL-100(Fe) was then evaluated. As indicated by XRD analysis (Figure 8a), the crystallinity of ABTS@MIL-100(Fe) was preserved following catalytic testing, while comparison of the characteristic FT-IR vibration bands of ABTS@MIL-100(Fe) with those of ABTS@MIL-100(Fe) after seven catalytic cycles confirmed the integrity of the ABTS unit inside the MIL-100(Fe) structure in addition to preservation of the crystalline structure (Figure 8b). Furthermore, the concentration of ABTS was estimated using its characteristic UV-Vis signal at ~340 nm (Figure 8c). Following the dispersion of ABTS@MIL-100(Fe) in water over a range of different time intervals, no color change was observed by direct macroscopy observations after either 10 h or 5 days. Indeed, after 10 h, virtually no ABTS was detected in the supernatant solution, indicating the ABTS leaching was not an issue in the short term. Furthermore, after 5 days, a negligible amount of ABTS was detected, thus further confirming that ABTS leaching was minimal.

## 3. Discussion

As described above, we successfully encapsulated ABTS into MIL-100(Fe) via a one-pot hydrothermal synthetic approach according to a similar previously reported method [26]. As ABTS can be oxidized into the steady radical cation ABTS^+•^ to give a color change from bluish green to green [5], the green color observed for ABTS@MIL-100(Fe) can be attributed to oxidation of the ABTS molecules present in the MOF to ABTS^+•^ by the Fe(III) centers of MIL-100(Fe). In addition, this one-pot synthetic approach allows the ABTS molecules to be immobilized in an appropriate MOF pore size, thus providing wider options for ABTS encapsulation into MOFs when compared to simple adsorption processes. Therefore, this direct synthetic route can significantly decrease the risk of any ABTS rapid leaching. In addition, analysis of ABTS@MIL-100(Fe) by EDS indicated an S content of 1.33 wt %, thereby suggesting an ABTS content of ~0.1 mmol/g in the solid ABTS@MIL-100(Fe) sample. An ABTS/FeCl_3_·6H_2_O weight ratio of approximately 1:7 could thus be calculated, which agrees with the experimental stoichiometry employed during sample preparation. For higher weight ratios, an excess of ABTS^+•^ radical cations is observed in solution. The obtained UV-Vis and FT-IR spectra also confirmed the successful modification of the MIL-101(Fe) nanoparticles with ABTS. In addition, the N_2_-adsorption isotherm showed that ABTS-MIL-100(Fe) maintained the characteristic mesoporous solid structure of MIL-101(Fe), due to the presence of both mesoporous cages and microporous windows, which also confirmed that the MIL-100(Fe) framework remained unaltered upon ABTS loading. The specific surface of MIL-100(Fe) is lower than that reported in literature owing to the difference between the two synthesis methods of MIL-100(Fe) [26]. The as-synthesized MIL-100(Fe), in this paper, may contain much free unreacted trimesic acid linker, leading to low specific surface of MIL-100(Fe). The similar phenomenon had been observed by other investigators [25].

This strong immobilization of the ABTS molecules within MIL-100(Fe) can therefore be accounted for by 3 main factors. Firstly, the size of the hexagonal MIL-100(Fe) windows corresponds well to the size of ABTS [26]. Secondly, sufficiently strong ionic interactions exist between the SO_3_^−^ moieties of ABTS and the Fe^3+^ centers of MIL-100(Fe), as reported previously for sulfonic acid-functionalized MIL-101(Cr) bearing coordinatively unsaturated sites [38]. Thirdly, weak interactions such as electrostatic attractions and van der Waals forces may also play a role. These interactions can therefore prevent the rapid leaching of ABTS during use.

It was also necessary to ascertain that ABTS immobilized in the pores of MIL-100(Fe) could still serve as a redox mediator of laccase. As indicated in the cyclic voltammetry results shown in Figure 4, the electroactivity of ABTS was maintained in the microenvironment of the MIL-100(Fe), which implies that immobilized ABTS can indeed act as a laccase mediator. However, we did observe a slight shift in the optimum reaction pH, likely due to a pH of 5.5 favoring electron transfer between the enzyme, the MOFs, and the dye molecules. These results indicate that although ABTS was immobilized in MIL-100(Fe), it maintained its activity in the mediation of dye decolorization by laccase. Indeed, a similar phenomenon was observed in our previous study [32]. Moreover, although the laccase and free mediator system presents a minimal mass-transfer resistance to the substrates, a degree of mass-transfer resistance exists between laccase, the immobilized mediator, and the substrates, thus resulting in a slightly longer decolorization time for ABTS@MIL-100(Fe) compared to that of the free mediator. However, the immobilized mediator exhibits the key advantage of facile recycling, which is not possible with the free mediator.

**Figure 9:**
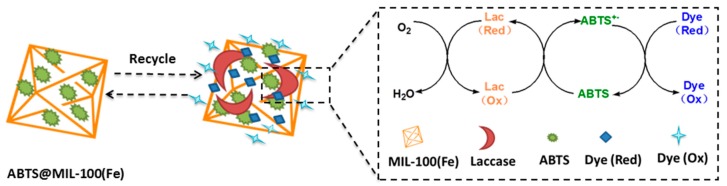
Schematic representation of the mechanism involved in dye decolorization via ABTS@MIL-100(Fe)-mediated laccase oxidation.

As mentioned previously, laccase belongs to the multicopper oxidase family and its substrate oxidation mechanism involves electron transfer [2]. In this context, the role of ABTS as a mediator in the enzymatic oxidation reaction has been previously described in the literature [39,40]. The encapsulation of laccase in the porosity of MIL-100(Fe) can be ruled out due to the diameter of the mesoporous cages of MOF. However, the enzyme can be adsorbed at the outer surface of MIL-100(Fe) nanoparticles via the repulsive electrostatic interactions. The fact that ABTS entrapped in the MIL-100(Fe) matrix can be catalyzed by laccase had been proved via the electrochemical impedance spectroscopy measurement [26]. As shown in Figure 9, ABTS immobilized in MIL-100(Fe) is first converted into the ABTS^+•^ radical cation via an enzymatic reaction, where molecular oxygen to is reduced to form water. Subsequently, the ABTS^+•^ radical cation takes part in non-enzymatic reactions with the dye species, reducing ABTS^+•^ to ABTS, and oxidizing the dye to complete a single catalytic cycle. This ultimately results in complete decolorization of the dye molecules. In our system, the immobilized ABTS effectively serves as an electron carrier to facilitate oxidation of the dye. On the other hand, Fe(III) in MIL-100(Fe) may be involved in the ABTS-laccase enzymatic reaction since it was previously reported that Fe(III) MOFs are involved in the formation of ^•^OH radical by catalyzing the degradation of H_2_O_2_ through a Fenton-type reaction [41].

Due to the potential economic benefits, the recovery and reuse of mediators is of particular importance upon the application of the enzyme/mediator system to industry or environmental protection on a large scale. Although ~74% decolorization was obtained following the seventh reuse cycle (Figure 7a), the decolorization efficiency gradually decreased upon increasing the number of cycles, which was attributed to both a loss of ABTS@MIL-100(Fe) during the recovery process and pore blockage by trapped dye degradation products, as previously reported for the catalyzing alcoholysis of styrene oxide by HPW@MIL-101 [39]. Indeed, although the production cost of ABTS@MIL-100(Fe) is slightly higher than that of the free mediator, the immobilized mediator can be easily reused, with large scale application of a reusable mediator likely lowering the relative costs associated with the immobilized mediator.

If dye removal was caused by biodegradation, it would be expected that the major visible light absorption peak would disappear completely [40], thus suggesting that the disappearance of the absorbance peak at 615 nm was related to the breakdown of the chromophoric group present in the dye. Furthermore, the decrease in peak intensity at 288 nm indicated breakdown of the unsaturated double bonds present in the dye [42]. Indeed, it has previously been reported that the chromophores and the benzene ring of the dye were degraded, thus resulting in detoxification of the dyes by preventing the formation of aromatic amines [43].

Finally, in addition to a high catalytic efficiency, both a high stability and a low level of leakage are important characteristics for the immobilized mediator. In this context, we confirmed the stability of ABTS@MIL-100(Fe) after seven catalytic cycles by XRD and FT-IR analysis. In addition, the low ABTS leaching and the high stability of ABTS@MIL-100(Fe) (as mentioned above) were attributed to the excellent chemical stability of MIL-100(Fe), which prevented the rapid release of ABTS molecules from the structure. This system also appears suitable for the treatment of dye wastewater due to the reported long-term stability of MIL-100(Fe) in water over two months [27].

## 4. Materials and Methods

### 4.1. Reagents and Materials

2,2′-Azino-bis-(3-ethylbenzthiazoline-6-sulphonate) (ABTS, C_18_H_24_N_6_O_6_S_4_) and trimethyl 1,3,5-benzenetricarboxylate (T-BTC, C_12_H_12_O_6_) were purchased from Sigma-Aldrich (St. Louis, MO, USA). Indigo carmine (C_16_H_8_N_2_Na_2_O_8_S_2_) and ferric chloride (FeCl_3_·6H_2_O) were obtained from Aladdin Bio-chem Technology (Shanghai, China). Laccase (enzyme activity ≥0.6 U/mg) was purchased from Sunson Industry Group (Beijing, China). All chemicals were of analytical grade and were used as received without further purification. Deionized water was used throughout.

### 4.2. Synthesis of the MIL-100(Fe) and ABTS@MIL-100(Fe) Nanoparticles

The ABTS@MIL-100(Fe) nanoparticles were prepared using a modified literature procedure [27]. In a typical synthesis, FeCl_3_·6H_2_O (0.189 g) was dissolved in water (20 mL), after which, the desired quantity of ABTS (i.e., ABTS/FeCl_3_·6H_2_O weight ratio = 1:1, 1:3, 1:5, 1:7, or 1:9) was added to determine the optimum quantity required for successful ABTS encapsulation into MIL-100(Fe). Following the addition of triethyl 1,3,5-benzenetricarboxylate (0.136 g), the mixture was heated at 130 °C for 72 h in a 50 mL Teflon-lined stainless steel bomb. The resulting green solid was then recovered by centrifugation and washed with water, ethanol, and diethyl ether to remove any unanchored ABTS and unreacted substrate. Finally, the green precipitate was dried under vacuum at 50 °C for 24 h. As an excess of ABTS was observed in solution for weight ratios of 1:1, 1:3, and 1:5, an ABTS/ FeCl_3_·6H_2_O weight ratio of 1:7 was employed for further synthesis and characterization. The MIL-100(Fe) nanoparticles were synthesized using the above-mentioned method but without the addition of ABTS.

### 4.3. Dye Decolorization

Indigo carmine dye was selected as a model substrate for the decolorization reaction. The effect of pH on dye decolorization by laccase using both ABTS@MIL-100(Fe) and ABTS was examined between pH 3.0 and 6.0 (in 0.05 M acetate buffer). In all experiments, the decolorization conditions were as follows: 50 mg/L dye, 2000 U/L laccase, and either the immobilized mediator (0.25 g/L for ABTS@MIL-100(Fe)) at pH 5.5 or the free mediator (25 µM for ABTS) at pH 4.5 (in 0.05 M acetate buffer). All experiments were carried out in 5 mL centrifuge tubes, and the mixtures were shaken at 150 rpm at 25 °C. Control reactions were also performed to study the decolorization efficiency by laccase and ABTS@MIL-100(Fe) alone. The extent of decolorization was determined spectrophotometrically by monitoring the decrease in absorbance at the maximum absorption wavelength of the dye (i.e., 615 nm). The percentage decolorization of a dye after the desired time was calculated based on the formula: decolorization (%) = (A_0_ − A_1_)/A_0_, where A_0_ is the initial absorbance of the dye at its adsorption maximum, and A_1_ is its absorbance after the desired reaction time. Dye degradation was monitored by UV-Vis spectroscopy and changes in the absorbance spectrum of the dye between 200 and 800 nm were recorded.

### 4.4. Electrochemical Analysis

Electrochemical measurements were performed at 25 °C using a conventional three-electrode system. A Pt wire was used as the working electrode, an Ag/AgCl electrode was employed as the reference electrode, and a gold electrode was used as the counter electrode. The ABTS@MIL-100(Fe)-modified Pt electrode was prepared as follows. Firstly, ABTS@MIL-100(Fe) (1 mg/mL) was mixed with a few drops 0.5 wt % Nafion solution, and the resulting mixture was spread on the surface of the Pt electrode to prepare a thin film, which was allowed to dry at 40 °C for 2 h. A 0.05 M acetate buffer at pH 5.5 was used as the electrolyte solution. A computer controlled CHI660A electrochemical workstation was employed for all electrochemical experiments.

### 4.5. Reusability of ABTS@MIL-100(Fe)

The recyclability of ABTS@MIL-100(Fe) was assessed using a discontinuous decolorization reaction as described above. At the end of each cycle, the ABTS@MIL-100(Fe) particles were separated by centrifugation and washed three times with distilled water (3 mL). The recovered ABTS@MIL-100(Fe) was then added to a fresh reaction solution to begin the new cycle. The data presented herein are average values of measurements repeated in triplicate.

### 4.6. Characterization

FT-IR spectroscopy was carried out using a Tensor 27 spectrometer (Bruker, Karlsrohe, Germany) with the KBr pellet technique. Field emission SEM and EDS were carried out using a JSM 6700F (JEOL, Tokyo, Japan). XRD was carried out using a Bruker D8-Advance (Bruker) with Cu-Kα radiation, while CV measurements were performed using a CHI600E electrochemical workstation (CH Instruments, Austin, TX, USA). Finally, UV-Vis spectroscopy was carried out using a Varian CARY50 spectrophotometer (Varian, Palo Alto, CA, USA).

## 5. Conclusions

In summary, we successfully demonstrated that 2,2′-azino-bis(3-ethylbenzthiazoline-6-sulfonic acid) (ABTS), which is commonly employed as a redox mediator for oxidative enzymes, could be immobilized into a mesoporous metal-organic framework sample (i.e., MIL-100(Fe)) by encapsulation via a one-pot hydrothermal method. The formation of ABTS@MIL-100(Fe) was confirmed by Fourier transform infrared spectroscopy (FT-IR), scanning electron microscopy (SEM), X-ray diffraction (XRD), and energy dispersive spectroscopy (EDS). In addition, cyclic voltammetry studies confirmed that the immobilized ABTS exhibited a comparable electroactivity to that of the free ABTS molecule, suggesting that the immobilization of ABTS did not alter its redox properties. We also demonstrated that ABTS@MIL-100(Fe) was suitable for use as an efficient mediator of laccase for the decolorization of indigo carmine dye (94% decolorization after 50 min), and the stability of ABTS@MIL-100(Fe) was confirmed by XRD and FT-IR over seven catalytic cycles (74% decolorization). Furthermore, although a negligible amount of ABTS was detected in the supernatant after storing in water for 5 days, essentially no ABTS was detected after 10 h, thus confirming the stability of this system. Moreover, unlike the free mediator, the immobilized mediator was easily recovered and reused following dye decolorization, thus improving the efficiency of mediator utilization and reducing both costs and secondary pollution. These results therefore confirm that the use of ABTS@MIL-100(Fe) as an immobilized laccase mediator has potential for application in the treatment of dye wastewater.
